# ImReLnc: Identifying Immune-Related LncRNA Characteristics in Human Cancers Based on Heuristic Correlation Optimization

**DOI:** 10.3389/fgene.2021.792541

**Published:** 2022-01-10

**Authors:** Meihong Gao, Shuhui Liu, Yang Qi, Xinpeng Guo, Xuequn Shang

**Affiliations:** School of Computer Science and Engineering, Northwestern Polytechnical University, Xi’an, China

**Keywords:** immune-related lncRNA, pathogenicity levels, heuristic correlation coefficient, enrichment analysis, immune infiltration

## Abstract

Long non-coding RNAs (lncRNAs) play critical roles in cancer through gene expression and immune regulation. Identifying immune-related lncRNA (irlncRNA) characteristics would contribute to dissecting the mechanism of cancer pathogenesis. Some computational methods have been proposed to identify irlncRNA characteristics in human cancers, but most of them are aimed at identifying irlncRNA characteristics in specific cancer. Here, we proposed a new method, ImReLnc, to recognize irlncRNA characteristics for 33 human cancers and predict the pathogenicity levels of these irlncRNAs across cancer types. We first calculated the heuristic correlation coefficient between lncRNAs and mRNAs for immune-related enrichment analysis. Especially, we analyzed the relationship between lncRNAs and 17 immune-related pathways in 33 cancers to recognize the irlncRNA characteristics of each cancer. Then, we calculated the Pscore of the irlncRNA characteristics to evaluate their pathogenicity levels. The results showed that highly pathogenic irlncRNAs appeared in a higher proportion of known disease databases and had a significant prognostic effect on cancer. In addition, it was found that the expression of irlncRNAs in immune cells was higher than that of non-irlncRNAs, and the proportion of irlncRNAs related to the levels of immune infiltration was much higher than that of non-irlncRNAs. Overall, ImReLnc accurately identified the irlncRNA characteristics in multiple cancers based on the heuristic correlation coefficient. More importantly, ImReLnc effectively evaluated the pathogenicity levels of irlncRNAs across cancer types. ImReLnc is freely available at https://github.com/meihonggao/ImReLnc.

## 1 Introduction

Cancer is a major threat to human health with high incidence and mortality (Siegel et al., 2015; Bray et al., 2018; Ferlay et al., 2019; Gharib et al., 2019). In 2018, there were approximately 18.1 million new cases and 9.6 million cancer deaths worldwide (Bray et al., 2018; Ferlay et al., 2019). Although early detection and treatment of cancer have increased the number of survivors, the results are still limited (Miller et al., 2016, 2019). Previous studies have reported that there is a link between lncRNA and the occurrence of cancer, and lncRNA plays an important role in cancer by gene expression and immune regulation (Gibb et al., 2011; Yang et al., 2014; Jiang et al., 2017; Li et al., 2020; Gao et al., 2021). The exploration of the lncRNA regulation mechanism is of great significance to the diagnosis and treatment of cancer (Bhan et al., 2017; Gharib et al., 2019, 2021).

Long non-coding RNA (lncRNA) refers to a transcript located in the nucleus or cytoplasm and is more than 200 nucleotides in length and cannot encode protein. (Wang and Chang, 2011; Ransohoff et al., 2018; Nair et al., 2020). With the development of sequencing technology, more and more lncRNAs have been identified and annotated (Zhao et al., 2016; Frankish et al., 2021). Studies have shown that lncRNA is related to the occurrence of cancer and participates in cell life activities through epigenetic modification, transcriptional regulation, post-transcriptional processing, and translational regulation (Esteller, 2011; Bao et al., 2019; Chi et al., 2019; Zhao et al., 2020). For example, GATA6-AS epigenetically regulates endothelial gene expression by interacting with LOXL2 (Neumann et al., 2018), CCR5AS regulates the expression of CCR5 to affect the outcome of HIV infection (Kulkarni et al., 2019), and OIS1 regulates the activation of DPP4 to control RAS-induced senescence (Li et al., 2018). In addition, lncRNA is involved in various processes of immune response in the tumor microenvironment (TME) and plays an important immunomodulatory role in cancer. It is reported that lncRNA NKILA promotes tumor immune escape by sensitizing T cells to activation-induced cell death (Huang et al., 2018) and lncRNA HOTAIR regulates the expression of the glucose transporter Glut1 and glucose uptake in macrophages during inflammation (Obaid et al., 2021).

TME is a complex environment around tumors and is crucial for tumor growth, invasion, and metastasis (Quail and Joyce, 2013; Yuan et al., 2016). The composition of TME varies by tumor type, but hallmark features include immune cells, stromal cells, blood vessels, and extracellular matrix (Joyce and Fearon, 2015; Spill et al., 2016; Anderson and Simon, 2020). There is a close interaction between tumor cells and their surrounding TME, and the phenomenon of other cells entering the tumor tissue is called infiltration (Fridman et al., 2011). The infiltration of immune cells in tumor tissue significantly impacts tumor proliferation and metastasis. Lymphocytes that infiltrate tumor tissues are called tumor-infiltrating lymphocytes, which include T cells and B cells and are part of the larger category of tumor-infiltrating immune cells (Fridman et al., 2011; Nazemalhosseini-Mojarad et al., 2019). The abundance of tumor-infiltrating lymphocytes varies with tumor type and stage and is related to the prognosis of the disease in some cases (Fridman et al., 2011; Hanahan and Coussens, 2012; Coussens et al., 2013). Tumor-infiltrating lymphocytes can usually be found in tumor stroma and tumor itself, where they can help tumor cells escape and promote the development of malignant tumors. LncRNAs participate in tumor-stroma crosstalk and stimulate a distinctive and suitable tumor microenvironment (Sun et al., 2020; Zhou et al., 2020). Thus, it is necessary to identify immune-related lncRNA (irlncRNA) to explore the immune regulation mechanism of lncRNA in tumors.

Identifying irlncRNA helps us understand the mechanism of cancer at the molecular level, contributing to the diagnosis and treatment of cancer. However, due to lncRNA’s specific expression, its pathogenicity level across cancer types is difficult to estimate. The development of computational methods for identifying irlncRNA in cancer and assessing its pathogenicity can help solve this problem. Several methods have been proposed to identify irlncRNAs for human cancers (Shen et al., 2020; Sun et al., 2020; Zhang et al., 2020; Gao et al., 2021), but the common goal of these methods is to identify irlncRNAs associated with single cancer. There is only one method named ImmLnc that can identify irlncRNAs for multiple cancers (Li et al., 2020). ImmLnc has a good performance on the given cancer dataset, but it has two limitations that need to be improved. On the one hand, ImmLnc uses partial correlation to calculate the ranking score, but direct correlation and partial correlation coexist in cancer tissues. On the other hand, ImmLnc uses the number of cancers in which irlncRNA-pathway pair is involved and the number of cancers in which irlncRNA is differentially expressed to calculate the pathogenicity level of irlncRNA, but other factors may also affect the pathogenicity level of irlncRNA. Therefore, it is necessary to integrate direct correlation and partial correlation to identify irlncRNA for human cancers and consider adding other factors to evaluate the pathogenic level of irlncRNA effectively.

Here, we proposed a new method ImReLnc for two main objectives. Firstly, we used heuristic correlation coefficients to provide ranking scores for immune-related enrichment analysis to identify irlncRNAs in human cancers. Especially, we integrated direct correlation coefficients and partial correlation coefficients based on the logistic function to obtain the heuristic correlation coefficients. We identified the irlncRNAs of 33 cancers and found that these irlncRNAs were highly expressed in immune cells, showed expression differences in cancers, and were significantly related to immune infiltration levels. The second goal of this method is to evaluate the pathogenicity levels of the identified irlncRNAs. For each irlncRNA identified by ImReLnc, we calculated not only the number of cancers in which lncRNA-pathway pair was involved but also the number of cancers in which irlncRNA was differentially expressed and its average fold change in those differentially expressed cancers. By comparing irlncRNAs with disease-related lncRNAs (drlncRNAs) in LncRNADisease2.0 (Bao et al., 2018) and Lnc2Cancer2.0 (Gao et al., 2018), we found that highly pathogenic irlncRNAs appeared in a higher proportion of known disease database than low pathogenic irlncRNAs. This demonstrates that ImReLnc is a valuable resource for predicting the pathogenicity of irlncRNA in human cancers.

## 2 Materials and Methods

**FIGURE 1:**
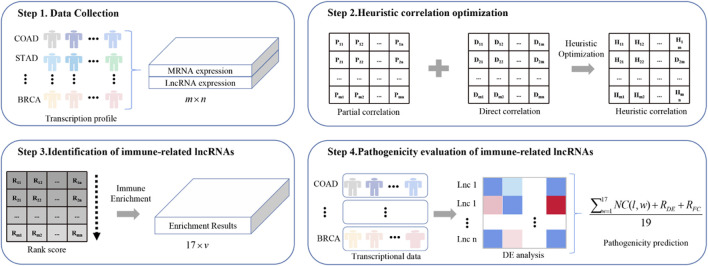
The flowchart of ImReLnc. The expression matrix in step 1 refers to the normalized expression profile of 33 cancers. The correlation in step 2 refers to the correlation between mRNA and lncRNA.

Figure 1 shows the framework of ImReLnc for identifying irlncRNAs and evaluating their pathogenicity levels.

### 2.1 Data Acquisition and Processing

#### 2.1.1 Expression Data Collection

The expression profiles of 33 types of cancer were downloaded from TCGA (See Supplementary Table S1, Data Sheet 1). For each type of cancer, we used Perl to process the expression profiles of all samples into an expression matrix, which was filtered by the annotation file in GENECODE (v22) to obtain the expression matrix of mRNA and lncRNA.

Hepatic carcinoma-related single-cell sequencing data was downloaded from Panglaodb. We performed cluster analysis on single-cell sequencing data to obtain the expression matrix of immune and non-immune cells through the Seurat package in R software. We further analyzed the expression matrix of lncRNA in these two types of cells and obtained the expression matrix of irlncRNA and non-irlncRNA in them.

#### 2.1.2 Immunization Data Collection

Immune-related gene lists were downloaded from Immport, which is one of the largest public databases for collecting and sharing immunology-related research resources. A total of 17 immune pathways and 1793 immune-related mRNAs were obtained for immune-related enrichment analysis, and the number of mRNAs associated with immune pathways ranged from 3 to 505 (See Supplementary Table S2, Data Sheet 1).

The immune infiltration levels of TCGA cancer samples (33 types of cancer samples) were downloaded from Timer. We obtained the infiltration levels of these cancer samples in six types of cells: B cell, T cell CD4^+^, T cell CD8^+^, Neutrophil, Macrophage, and Myeloid dendritic cell.

#### 2.1.3 LncRNA-Disease Interactions Collection

We collected the human lncRNA–disease interactions from LncRNADisease and Lnc2Cancer. Specifically, 2665 drlncRNAs were collected from Lnc2Cancer, and 6,105 drlncRNAs were collected from Lnc2Cancer. After preprocessing (removal of duplicate items), there were 2665 drlncRNAs left in Lnc2Cancer and 5,714 drlncRNAs left in LncRNADisease.

### 2.2 Heuristic Correlation Optimization

**FIGURE 2:**
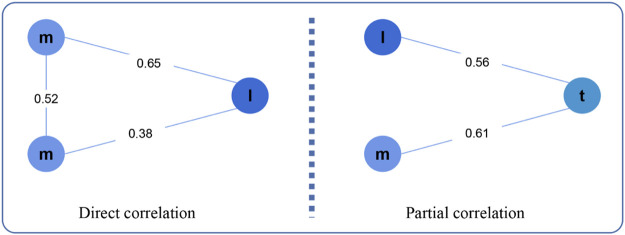
Direct correlation and Partial correlation. Here, m, l, t represents mRNA, lncRNA, tumor purity, respectively.

LncRNA can regulate the expression of mRNA to cause cancer, and there are two regulatory patterns: direct regulation and indirect regulation (Figure 2). In the first pattern, the expression values of lncRNA and mRNAlncRNA and mRNA expression values are directly related, while in the second pattern, they are partially related.

#### 2.2.1 Direct Correlation Between mRNAs and lncRNAs

The direct correlation was computed through the method of Pearson and Spearman (Benesty et al., 2009; Sedgwick, 2014). The Pearson’s rank correlation coefficient between mRNA *m* and lncRNA *l* is defined as follows:

$$R\left(m,l\right)=\frac{E\left(ml\right)-E\left(m\right)E\left(l\right)}{\sqrt{E\left(m^{2}\right)-E^{2}\left(m\right)}\sqrt{E\left(l^{2}\right)-E^{2}\left(l\right)}}$$ (1)

where function *E* is used to calculate the mathematical expectation of variables. Similarly, the Spearman’s correlation coefficient between mRNA *m* and lncRNA *l* is defined as follows:

$$S\left(m,l\right)=1-\frac{6\sum \overset{2}{\underset{i}{d}}}{r\left(r^{2}-1\right)}$$ (2)

where *d*_*i*_ represents the difference between the rank of *m* and *l*, and *r* represents the number of cancer samples. Although Pearson correlation and Spearman correlation can effectively fit the correlation between mRNA and lncRNA in cancer samples, they have some limitations. To make the value of direct correlation more accurate, we combined these two types of correlation coefficients to represent the direct correlation coefficient as follows:

$$D\left(m,l\right)=\frac{R\left(m,l\right)+S\left(m,l\right)}{2}$$ (3)

#### 2.2.2 Partial Correlation Between mRNAs and lncRNAs

Tumor purity affects the expression of mRNA and lncRNA in cancer samples. To eliminate this effect, we calculated the partial correlation between mRNA and lncRNA based on tumor purity. The tumor purity of the sample was calculated by analyzing its mRNA expression profile using the estimate package of R software. The partial correlation coefficient between mRNA *m* and lncRNA *l* in sample *t* is defined as follows:

$$P\left(m,l\right)\left(t\right)=\frac{D\left(m,l\right)-D\left(m,t\right)D\left(t,l\right)}{\sqrt{1-D^{2}\left(m,t\right)}\sqrt{1-D^{2}\left(t,l\right)}}$$ (4)

#### 2.2.3 Heuristic Correlation Between mRNAs and lncRNAs

Both direct and partial correlations play an important role in lncRNA regulating mRNA expression. Thus, we integrated these two correlations to calculate the heuristic correlation between mRNA and lncRNA. To highlight the strong correlation and to fade the weak correlation, we defined a logistic function as follows:

$$L\left(x\right)=\frac{1}{e^{cx+d}+1}$$ (5)

where *x* is the absolute value of the correlation coefficient. When *c* is equal to -15 and *d* is equal to ln (1999), *L*(*x*) is approximately equal to 0 in the interval [0, 0.3], and *L*(*x*) is approximately equal to 1 in the interval [0, 0.7] (See Supplementary Figure S1, Data Sheet 1). Furthermore, we define the heuristic correlation coefficient between mRNA m and lncRNA l as follows:

$$\begin{array}{c} H\left(m,l\right)\left(t\right)=L\left[\beta D\left(m,l\right)+\left(1-\beta \right)P\left(m,l\right)\left(t\right)\right] \end{array}$$ (6)

where *β* ranges from 0 to 1 and is limitid as follows:

$$\underset{\beta }{\mathrm{arg min}}\left\{1-|\beta D\left(m,l\right)+\left(1-\beta \right)P\left(m,l\right)\left(t\right)|\right\}$$ (7)

### 2.3 Identification of Immune-Related lncRNAs

#### 2.3.1 Immune-Related Enrichment Analysis

The immune regulation mechanism of lncRNA was explored by analyzing the relationship between lncRNA-related mRNA and immune pathways. First, we performed a screening on the heuristic correlation matrix to obtain the rank matrix *R*_*ML*_ (the filtering threshold is 0.5). As a result, *v* lncRNA-related mRNA classes were obtained. Each class was composed of heuristic correlation coefficients between a lncRNA and the corresponding *u* mRNAs and was sorted in descending order according to these coefficients. Then we conducted enrichment analysis for the lncRNA-related mRNA classes on 17 immune pathways using fgsea package in R software. The minimum and maximum sizes of the mRNA class were set to 1 and 5,000, respectively, and the number of permutations was set to 10 000. The enrichment analysis result was a 17*v* × 10 matrix, whose column information includes lncRNA, immune pathway, *p*-value, adjusted *p*-value, enrichment score, and five other enrichment parameters.

#### 2.3.2 Extraction of Immune-Related lncRNAs

We evaluated the relationship between lncRNA and immune pathway based on the enrichment score and *p* value as follows (Li et al., 2020):

$$lncRES\left(l,w\right)=\left\{\begin{array}{cc} 1-2p,\; & E\left(l,w\right)\geq 0\\ 2p-1,\; & E\left(l,w\right)<0 \end{array}\right.$$ (8)

where *p* represents the *p*-value of the enrichment result, and *E* (*l*, *w*) represents the enrichment score of lncRNA *l* on pathway *w*. Obviously, the range of *lncRES* (*l*, *w*) is [ − 1, 1], and the greater the absolute value, the higher the enrichment degree. For each type of cancer *c*, |*I*_*c*_| irlncRNAs were obtained by setting the absolute value of *lncRES* (*l*, *w*) to be greater than 0.995 and setting the FDR to be less than 0.05. Finally, the set of all irlncRNAs in 33 types of cancer can be defined as follows:

$$I=\overset{33}{\underset{c=1}{⋃}}I_{c}$$ (9)

Each irlncRNA in set *I* has several corresponding immune pathways to form lncRNA-pathway pairs, in which the lncRNA plays an immunomodulatory role in cancer by acting on the immune pathway.

### 2.4 Pathogenicity Evaluation of Immune-Related lncRNAs

After identifying cancer-related irlncRNAs, we further analyzed these irlncRNAs to explore their pathogenicity levels.

#### 2.4.1 Differential Expression Analysis of Immune-Related lncRNAs

Since the differential expression level of lncRNA greatly influences on the pathogenicity of lncRNA, we performed differential expression analysis of the identified irlncRNAs in 33 cancers by using the edger package of R software. Here, the fold change and the adjusted *p* values were used to evaluate the expression differences of irlncRNAs, and the differentially expressed irlncRNAs were acquired by setting the adjusted *p*-value < 0.01 and the absolute value of logFC > 1.5. *R*_*DE*_(*l*) is used to reflect the range of differential expression of irlncRNA *l* in set *I* and is defined as follows:

$$R_{DE}\left(l\right)=N\left[NC\left(l\right)\right]$$ (10)

where function *N* is used for normalization, and *NC*(*l*) represents the number of cancers in which irlncRNA *l* from set *I* is differentially expressed. The range of *NC*(*l*) is [0, 24], and the upper limit depends on the number of cancers (with tumor and normal samples) for which we performed differential expression analysis. *R*_*FC*_(*l*) is used to reflect the intensity of differential expression of irlncRNA *l* in set *I* and is defined as follows:

$$R_{FC}\left(l\right)=N\left[\frac{\sum_{c=1}^{NC\left(l\right)}FC\left(c,l\right)}{NC\left(l\right)}\right]$$ (11)

where function *N* is used for normalization, and *FC*(*c*, *l*) represents the fold change of the expression value of irlncRNA *l* in cancer *c*.

#### 2.4.2 Pathogenicity Prediction of Immune-Related lncRNAs

Because the number of cancers related to lncRNA-pathway pairs and the differential expression level of lncRNA have a great influence on the pathogenicity level of lncRNA, we merged these two kinds of information to evaluate the pathogenicity level of irlncRNA *l* in set *I* as follows:

$$Pscore\left(l\right)=\frac{\sum_{w=1}^{17}N\left[NC\left(l,w\right)\right]+R_{DE}+R_{FC}}{19}$$ (12)

where function *N* is used for normalization, and *NC*(*l*, *w*) represents the number of cancers in which lncRNA *l* regulates immune pathway *w*.

## 3 Results

We proposed a computational method, ImReLnc, to identify irlncRNAs and predict their pathogenicity levels across cancer types (Figure 1). The immunological and pathogenic properties of the identified irlncRNAs have been verified in this section. In particular, the experiment was performed on R 3.4.1 under Ubuntu 20.04.2, the CUDA version was 11.3, and the memory was 8 × 11019 M.

### 3.1 Immune-Related lncRNAs and Pathways

**FIGURE 3:**
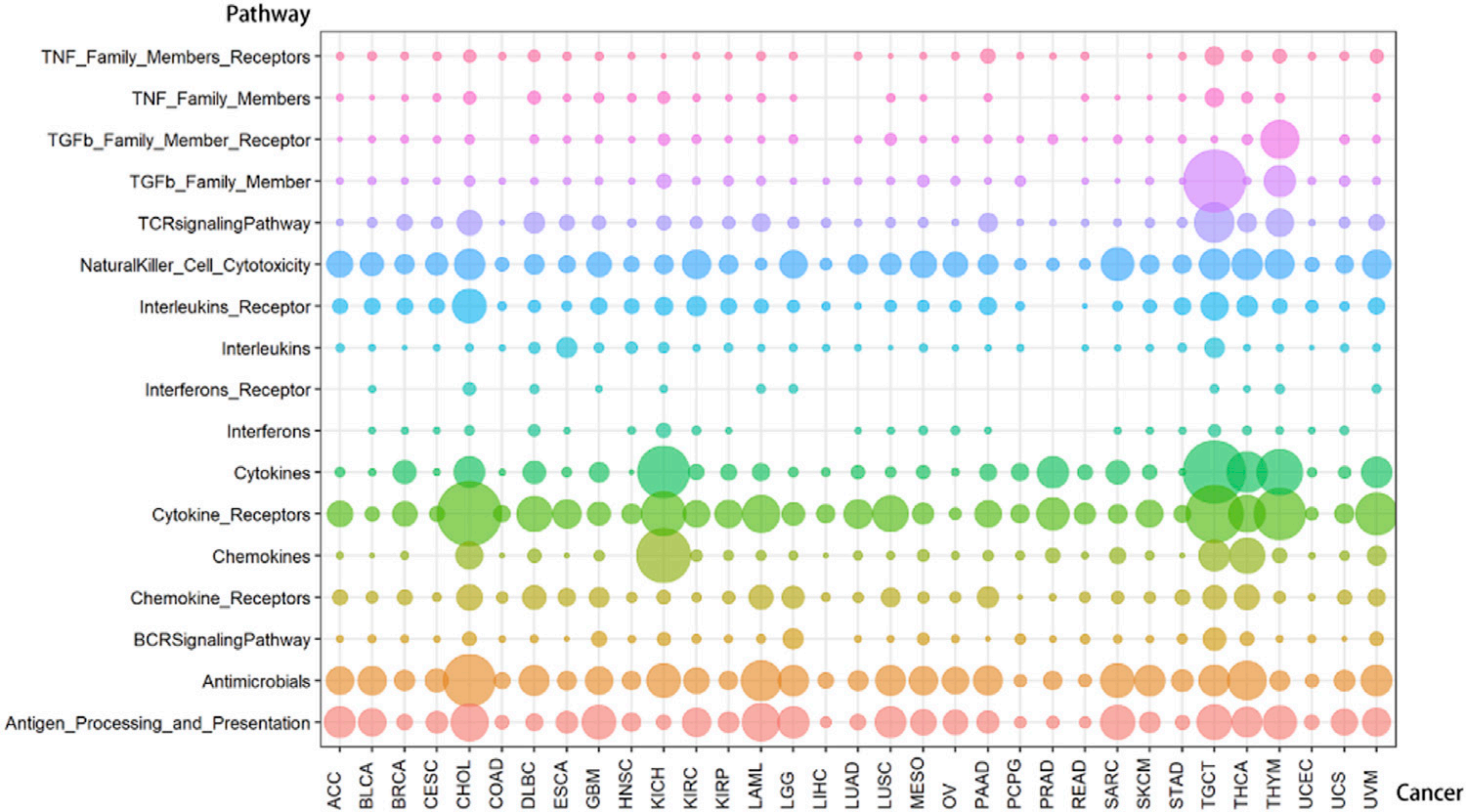
The distribution of lncRNA-pathway pairs in cancers. The lncRNAs in the lncRNA-pathway pairs refer to the immune-related lncRNAs (irlncRNAs), and the pathways in the lncRNA-pathway pairs refer to the immune pathways. Bubbles with different colors indicate the distribution of lncRNA-pathway pairs in different immune pathways, and the size of the bubbles is proportional to the number of irlncRNAs.

**FIGURE 4:**
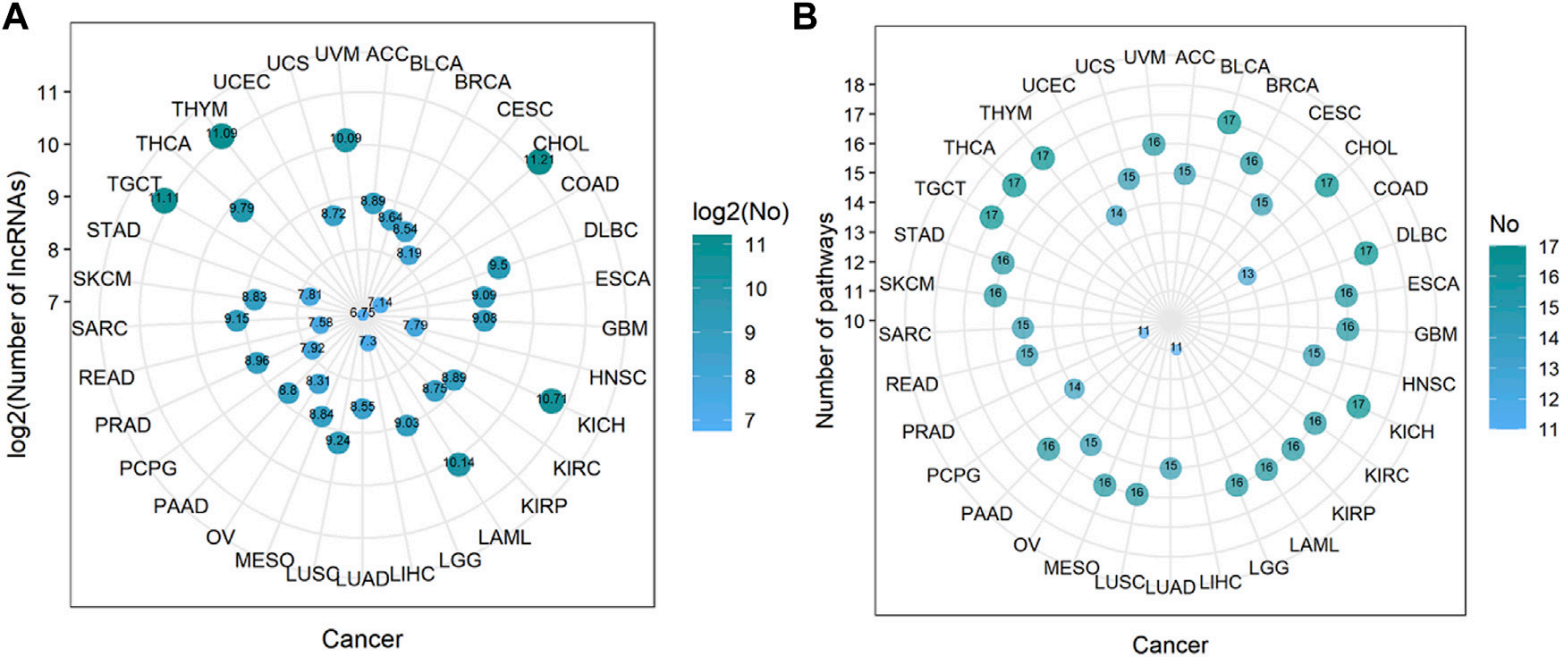
The number of immune-related lncRNAs and immune pathways in cancer. **(A)** The log value of the number of immune-related lncRNAs in cancer. The size of the bubbles is proportional to the log value of the number of immune-related lncRNAs. **(B)** The number of immune pathways in cancer. The size of the bubble is proportional to the number of immune pathways.

Through steps one to three in Figure 1, we comprehensively analyzed the heuristic correlation between mRNA and lncRNA of 33 cancers and the enrichment of lncRNA in 17 immune pathways. As a result, we obtained a series of lncRNA-pathway pairs (Figure 3) for each cancer. We found that the number of irlncRNA involved and the enriched immune pathways differed between cancers, and the degree of enrichment of cancers in some immune pathways tended to be similar. We further calculated the number of cancer-related irlncRNAs (Figure 4A) and immune pathways (Figure 4B). It was found that the number of cancer-related irlncRNAs ranged from 6.75 to 11.21 (after log2). Specifically, the number of UCEC-related irlncRNAs was the least at 6.75 (after log2), and the number of CHOL-related irlncRNAs was the highest at 11.21 (after log2). As for the number of cancer-related immune pathways, they ranged from 11 to 17. It should be noted that among 33 types of cancers, two types (6.1%) were enriched in 11 pathways, one type (3%) was enriched in 13 pathways, two types (6.1%) were enriched in 14 pathways, eight types (24.2%) were enriched in 15 pathways, thirteen types (39.4%) were enriched in 16 pathways, and the remaining seven types (21.2%) were enriched in 17 pathways. We found that the number of cancer types enriched in more than 16 pathways accounted for 60.6% of the total cancer types, and the number of cancer types enriched in more than 15 pathways accounted for 84.8% of the total cancer types. This indicates that most of the pathways are involved in the immune regulation process of cancer.

**FIGURE 5:**
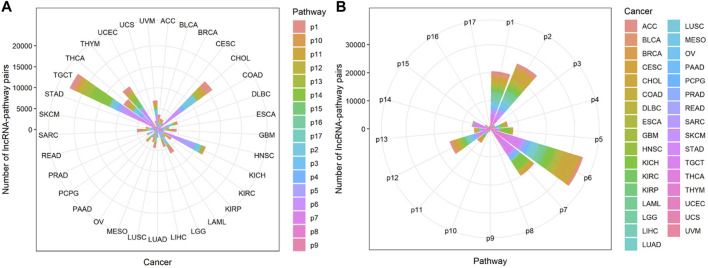
The enrichment of lncRNA-pathway pairs in cancers and immune pathways. The p1-17 represents Antigen Processing and Presentation, Antimicrobials, BCRSignalingPathway, Chemokine Receptors, Chemokines, Cytokine Receptors, Cytokines, Interferons, Interferons Receptor, Interleukins, Interleukins Receptor, NaturalKiller Cell Cytotoxicity, TCRsignalingPathway, TGFb Family Member, TGFb Family Member Receptor, TNF Family Members, and TNF Family Members Receptors respectively. **(A)** The Enrichment of lncRNA-pathway pairs in cancers. **(B)** The Enrichment of lncRNA-pathway pairs in immune pathways.

Compared with cancers with a small number of lncRNA-pathway pairs, cancers with a large number of lncRNA-pathway pairs are more likely to be enriched in the immune pathway, and for the convenience of description, we refer to these cancers as immune-preferred cancers in the following text. We analyzed the enrichment of cancers in immune pathways to find immune-preferred cancers (Figure 5A). Cholangiocarcinoma (CHOL), Kidney Chromophobe (KICH), Testicular Germ Cell Tumors (TGCT), Thyroid carcinoma (THCA), and Thymoma (THYM) were found to have more obvious immune enrichment, thus they are the so-called immune-preferred cancers. Studies have shown that immunotherapy plays an important role in the treatment of these five types of cancer (Schott et al., 2001; Drake and Stein, 2018; Høgdall et al., 2018; Jakopovic et al., 2020; Kalavska et al., 2020). For these cancers, immunotherapy can achieve better prognostic effects, and our findings can provide a reference for their immunotherapy.

**FIGURE 6:**
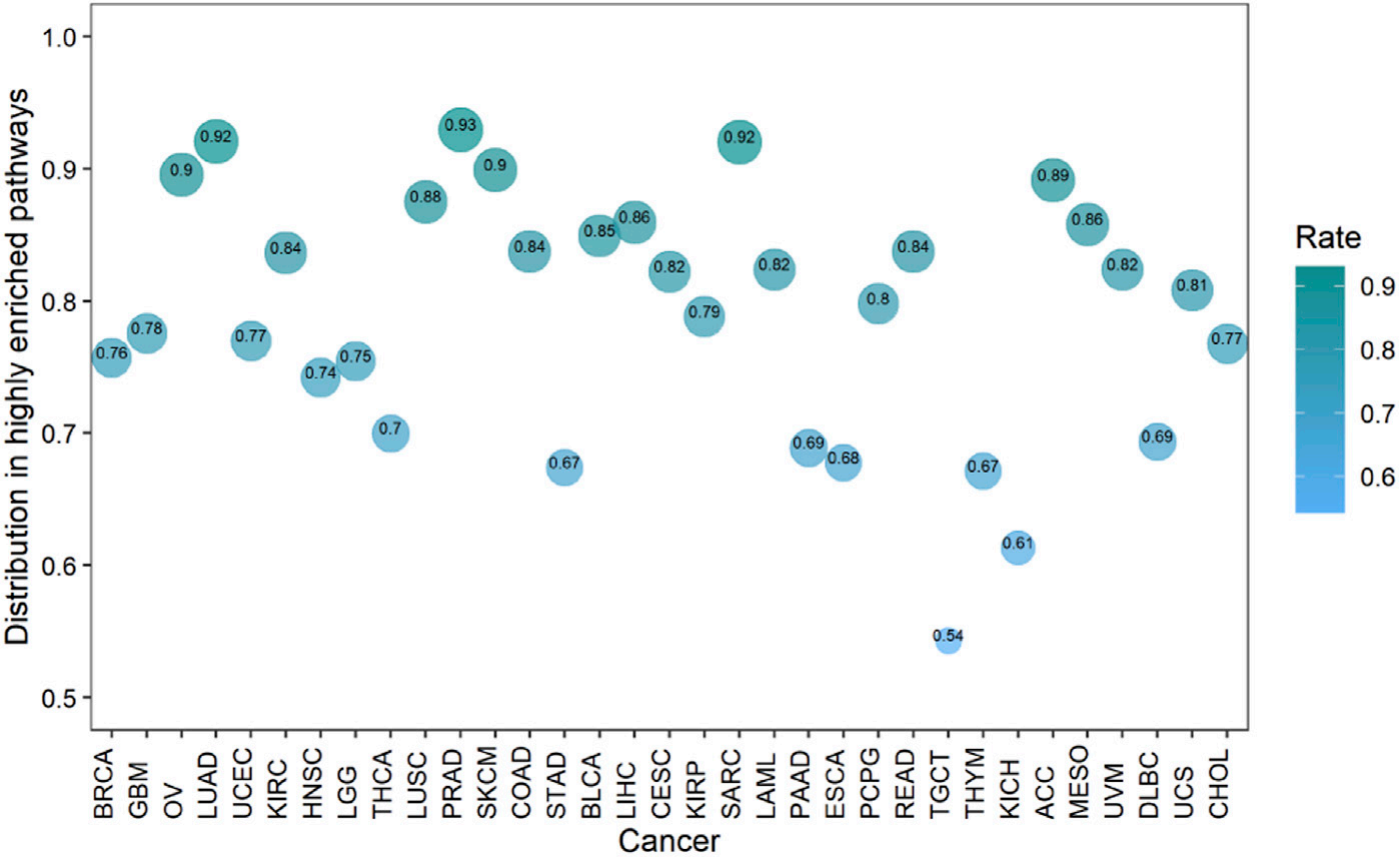
The enrichment of cancers in the highly enriched immune pathways. The size of the bubbles is proportional to the ratio of cancer in the highly enriched immune pathway.

Cancers also have a preference for their enriched immune pathways. They will be mainly enriched in some immune pathways, which we call highly enriched immune pathways, while their enrichment in other pathways will be very limited. We analyzed the distribution of lncRNA-pathway pairs on the immune pathways to discover highly enriched immune pathways in cancers (Figure 5B). Cancers were mainly enriched in 5 immune pathways (Antigen Processing and Presentation, Antimicrobials, Cytokine Receptors, Cytokines, and NaturalKiller Cell Cytotoxicity), and the average enrichment ratio of lncRNA-pathway pairs related to these pathways was 0.790 832 3. Specifically, 18 of 33 (more than half) cancers were enriched in these five immune pathways by more than 0.790 832 3 (Figure 6). Obviously, these five pathways are highly enriched immune pathways for those 33 cancers, and these pathways are active in the immune regulation process of the cancers. Antigen processing and presentation is involved in the decomposition and presentation of antigen proteins in immune regulation (Vyas et al., 2008). Antimicrobials are related to the life activities of bacteria (Van Harten et al., 2018). Cytokine Receptors and Cytokines play a signal transduction role in immune regulation (O’Shea et al., 2019). As for NaturalKiller Cell Cytotoxicity, it is related to the immune regulation of NaturalKiller Cell (Topham and Hewitt, 2009). The functions mentioned above are related to the life activities of cancer cells, thus the high enrichment of these five immune pathways is closely related to the occurrence of cancer.

### 3.2 The Distribution of Immune-Related lncRNAs

Cancers are enriched in immune pathways with a series of lncRNA-pathway pairs, and each pathway is related to a variety of lncRNAs which are the irlncRNAs to be recognized. These irlncRNAs are involved in the immune regulation process of cancer. We analyzed the characteristics of cancers and immune pathways related to them (See Supplementary Table S1, 2). It was found that the total number of irlncRNAs was 7,038, the number of cancers related to irlncRNA ranged from 1 to 32, and the number of immune pathways associated with irlncRNA ranged from 1 to 15. For the number of cancers and pathways in these ranges, we further explored the distribution of irlncRNAs on them (See Supplementary Figure S2, Data Sheet 1). Firstly, for the distribution of different numbers of cancers, we found that irlncRNAs related to only one type of cancer accounted for 0.417 448 1 of the total irlncRNAs, irlncRNAs related to two types of cancer accounted for 0.209 718 7 of the total irlncRNAs, irlncRNAs related to three types of cancer accounted for 0.114 237 of the total irlncRNAs, and the remaining 4–32 types of cancer-related irlncRNAs accounted for 0.258 596 2 of the total irlncRNAs. Then, for the distribution of different numbers of immune pathways, we found that irlncRNAs associated with one to four immune pathways accounted for 0.638 107 4 of the total irlncRNAs, and the remaining 0.361 892 6 of irlncRNAs were related to 5–15 pathways. Overall, more than half of irlncRNAs played a regulatory role in several types of cancer, and these regulatory roles involved multiple immune pathways.

The immunomodulatory effect in immune-preferred cancers is quite active, and we further analyzed irlncRNAs related to these cancers (See Supplementary Table S3). There were 34 irlncRNAs related to all five immune-preferred cancers, and another 5,097 irlncRNAs were related to one to four of the five immune-preferred cancers. The highly enriched immune pathway plays an important role in the immune regulation of cancers. Thus we obtained irlncRNAs related to the highly enriched pathways (See Supplementary Table S4). There were 1,264 irlncRNAs related to all five highly enriched immune pathways, and another 5,473 irlncRNAs were related to one to four of the five highly enriched immune pathways. Obviously, most irlncRNAs (0.627 558) were involved in one of the five immune-preferred cancers, and they were almost evenly distributed in the five pathways. This was significantly different from the distribution of irlncRNAs in total cancers and total immune pathways due to the immune preference of the cancers and the high enrichment of the immune pathways.

### 3.3 The Expression of Immune-Related lncRNAs

**FIGURE 7:**
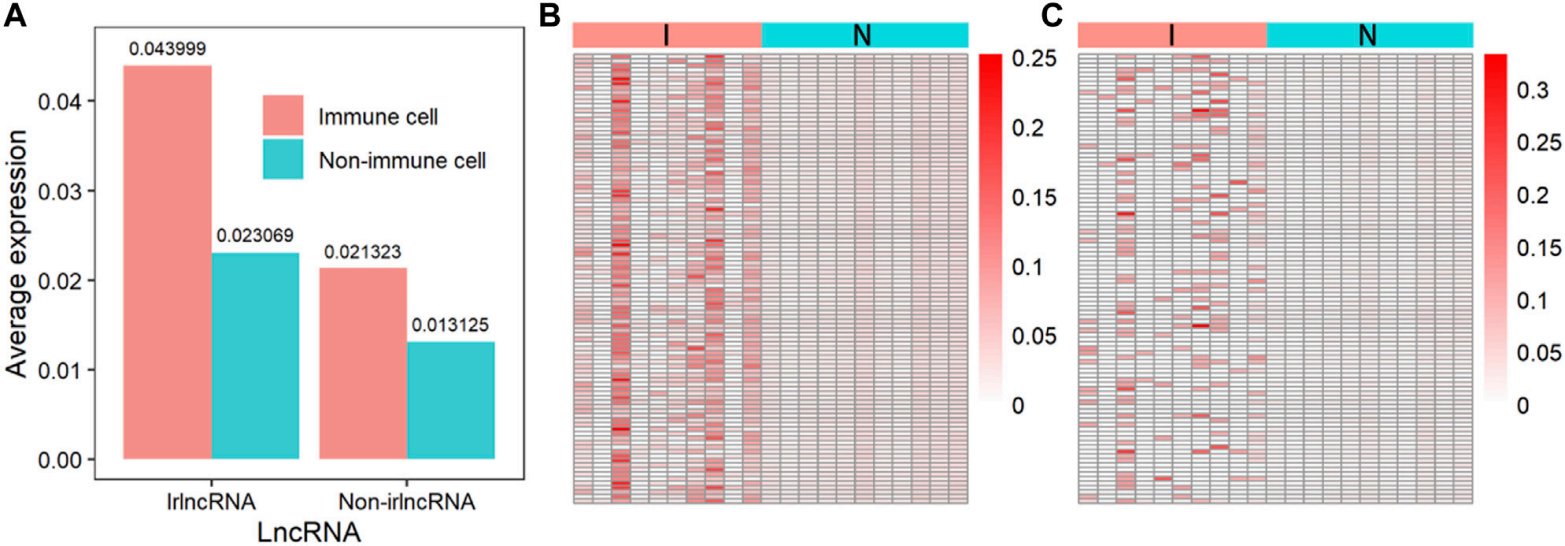
The expression of immune-related lncRNA (irlncRNA) and non-immune-related lncRNA (non-irlncRNA) in immune and non-immune cells. **(A)** The average expression of irlncRNA and non-irlncRNA in immune cells and non-immune cells. **(B)** The distribution of irlncRNA and non-irlncRNA in immune cells (*p*-value ≤0.01). **(C)** The distribution of irlncRNA and non-irlncRNA in non-immune cells (*p*-value ≤0.01).

Since the immune regulation process is performed by immune cells, the expression of irlncRNA in immune cells should be higher than that in other cells. We compared the expression levels of irlncRNA and non-irlncRNA in immune and non-immune cells on single-cell sequencing data from the PanglaoDB database. 1) We normalized the single-cell sequencing data of hepatic carcinoma. 2) The lncRNA count data was extracted. 3) The irlncRNA count data and non-irlncRNA count data were extracted. 4) We compared the expression of irlncRNA and non-irlncRNA in immune and non-immune cells, respectively (Figures 7A–C). It was found that the average expression of irlncRNA in immune cells was higher than that of non-irlncRNA, while in non-immune cells, their difference was slight (Figure 7A). The distributions of irlncRNA and non-irlncRNA in immune cells and non-immune cells further confirmed this conclusion (Figures 7B,C). To further explore the expression characteristics of irlncRNA, we analyzed the correlation between lncRNA expression and the level of immune infiltration in cancers. We first estimated the immune infiltration level of cancer samples through the TIMER online tool (Newman et al., 2015; Becht et al., 2016; Li et al., 2016; Aran et al., 2017; Finotello et al., 2019). Then, we calculated the correlation between lncRNA expression and the level of immune infiltration to find out infiltration-related lncRNAs (infrlncRNAs). Finally, we compared the distribution of our method (ImReLnc) and ImmLnc on irlncRNA and infrlncRNA. Specifically, we calculated the irlnRNA rate (IR) in all lncRNAs, the irlnRNA rate in infrlncRNA (IRINF), the infrlncRNA rate (INFR) in all lncRNAs, the infrlncRNA rate in irlncRNA (INFRI) for ImReLnc and ImmLnc (See Supplementary Table S3, S4, Data Sheet 1). It was found that the IRINF/IR and IRINF/IR of ImReLnc were significantly higher than ImmLnc in all 33 cancers (See Supplementary Figure S3, Data Sheet 1). Therefore, the irlncRNAs recognized by ImReLnc have more significant immune properties than those recognized by ImmLnc.

**FIGURE 8:**
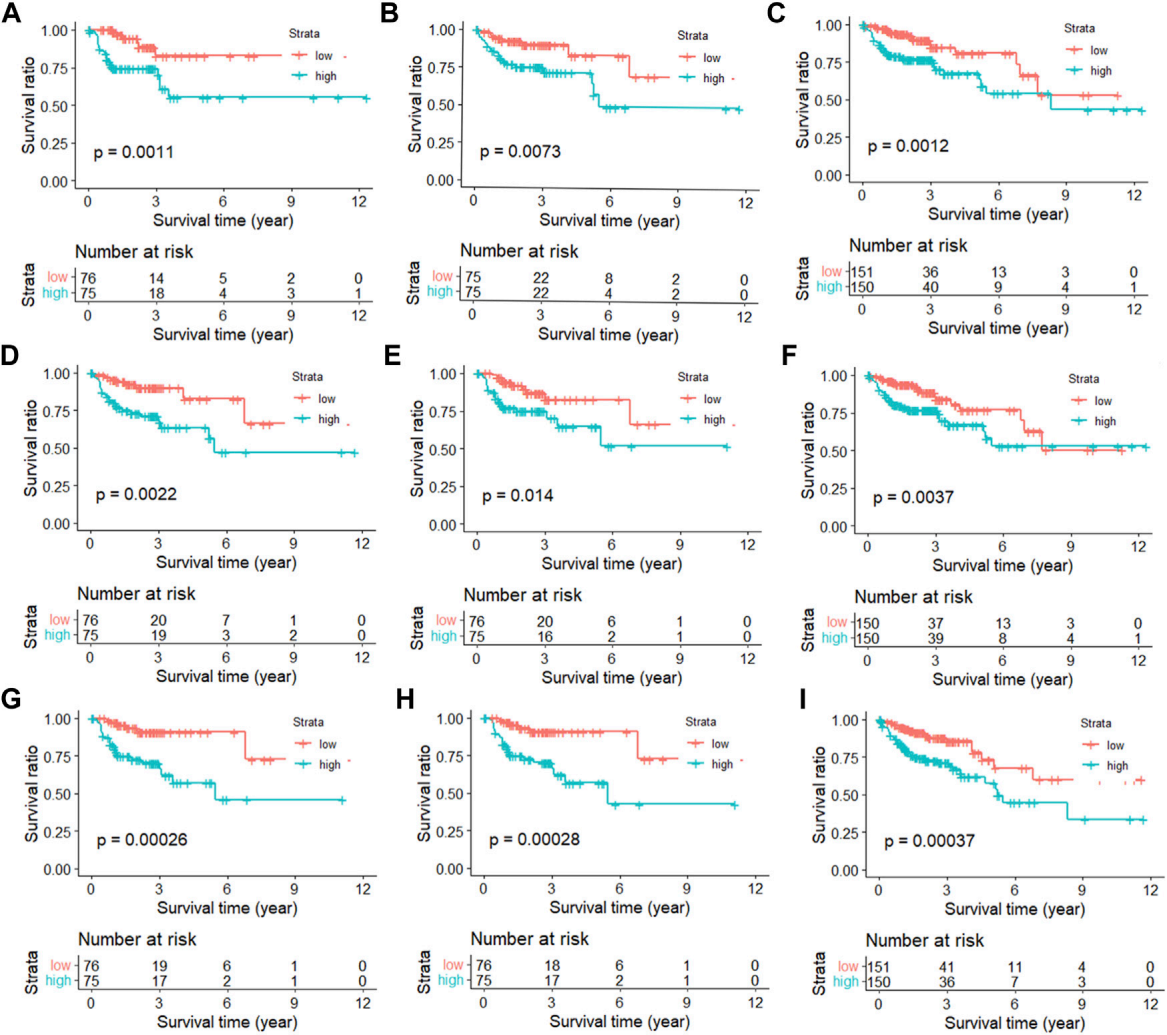
Kaplan-Meier curves of immune-related lncRNAs from different correlation calculations. **(A)** Direct correlation calculation in the training set. **(B)** Direct correlation calculation in the testing set. **(C)** Direct correlation calculation in the total set. **(D)** Partial correlation calculation in the training set. **(E)** Partial correlation calculation in the testing set. **(F)** Partial correlation calculation in the total set. **(G)** Heuristic correlation calculation in the training set. **(H)** Heuristic correlation calculation in the testing set. **(I)** Heuristic correlation calculation in the total set.

In order to show that the heuristic correlation coefficient is better than the direct or partial correlation coefficient, we performed the following analysis on the colon cancer data set. First, we calculated three correlation coefficients: direct correlation coefficient, partial correlation coefficient, and heuristic correlation coefficient. Then, we performed immune-related enrichment analysis based on the above correlation coefficients. Afterward, we screened the enrichment results and obtained three sets of irlncRNAs. Finally, we performed Cox regression analysis on the three groups of irlncRNAs. Specifically, six highly pathogenic irlncRNAs RP5-884M6.1, RP11-742B18.1, HOTAIR, AC004988.1, CTD-2357A8.3, and GS1-600G8.5, were found to be associated with cancer prognosis (Table 1). Among them, RP5-884M6.1, RP11-742B18.1, and HOTAIR were found to be related to the occurrence of colon cancer by previous studies (Zhou et al., 2019; Paredes et al., 2020; Gong et al., 2021). The prognostic analysis related to the three correlation calculations is shown in Figure 8. We found that the prognostic effects of lncRNA calculated by direct correlation and partial correlation were not much different. The prognostic effect of lncRNA calculated by heuristic correlation was better than the former two. This indicates that heuristic correlation calculation has advantages in identifying immune-related prognostic characteristics in cancer.

**TABLE 1:** Univariate and Multivariate Cox analysis of immune-related lncRNAs.

LncRNA	Univariate		Multivariate			Source
	HR (95% CI for HR)	p.value	Coef	z	p	
HOTAIR	0.97 (0.95–0.99)	0.026	5.79E-03	1.914	0.001 58	PMID: 34 630 664
AC004988.1	1 (1–1)	0.000 19	-4.88E-04	-1.638	0.001 66	-
RP5-884M6.1	1 (1–1)	0.001 9	1.83E-03	2.92	0.003 56	PMID: 32 983 990
CTD-2357A8.3	0.97 (0.95–0.99)	0.007 2	-0.001 200 4	-1.387	0.015 35	-
GS1-600G8.5	0.99 (0.98–1)	0.012	1.19E-02	1.154	0.029 29	-
RP11-742B18.1	1 (1–1)	0.000 98	-3.10E-02	-2.045	0.040 83	PMID: 31 516 583

### 3.4 The Pathogenicity Level of Immune-Related lncRNAs

Having proved that ImReLnc can effectively identify irlncRNA, we next explored the application of ImReLnc in assessing the pathogenicity level of irlncRNA. We first compared the relationship between drlncRNAs from disease databases (LncRNADisease2.0 and Lnc2Cancer2.0) and irlncRNAs recognized by ImmLnc and ImmLnc. Specifically, the top 500 irlncRNAs (determined by the number of cancers involved in irlncRNA) were used for analysis, and we found that the irlncRNA recognized by ImReLnc accounted for a higher proportion in the disease database (Table 2). Besides, we analyzed the distribution of pathogenicity levels of irlncRNAs in ImReLnc. Two methods were used to divide the data set where the sorted irlncRNAs (descending order) were located. The first method was to evenly divide irlncRNAs into 6 data sets according to the number of irlncRNAs (Table 3), and the second method was to divide irlncRNAs into 5 data sets evenly according to the level of pathogenicity of irlncRNAs (Table 4). In Table 3, the average pathogenicity level of irlncRNAs in LncRNADisease2.0 and Lnc2Cancer2.0 is higher than or approximately equal to that of all identified irlncRNAs, especially in Dataset 1. This indicates that the calculated pathogenicity level of irlncRNA is credible, and irlncRNA is particularly pathogenic within the range of high pathogenicity levels. Similarly, in the range of high pathogenicity levels in Table 4, the mean pathogenicity level of irlncRNAs in LncRNADisease2.0 and Lnc2Cancer2.0 is higher than the mean pathogenicity level of all identified irlncRNAs. This further shows that the calculated pathogenicity level of irlncRNA is credible, and its pathogenicity within the interval of high pathogenicity level is quite apparent.

**TABLE 2:** The comparison of the relationship between irlncRNA and drlncRNA in ImmLnc and ImReLnc. The data set in the table is divided equally according to the number of irlncRNAs. IrlncRNAs refer to immune-related lncRNAs, drlncRNA refers to disease-related lncRNAs.

Dataset	Distribution of ImReLnc			Distribution of ImmLnc		
	Count	LncRNADisease2.0	Lnc2Cancer2.0	Count	LncRNADisease2.0	Lnc2Cancer2.0
Total	500	110	111	500	106	109
Dataset1	100	29	25	100	27	22
Dataset2	100	27	29	100	28	25
Dataset3	100	30	31	100	25	29
Dataset4	100	14	11	100	15	20
Dataset5	100	10	15	100	11	13

**TABLE 3:** The pathogenicity distribution of immune-related lncRNA (irlncRNA). The data set is divided equally according to the number of irlncRNAs.

Pathogenicity level of IrlncRNA	Total	Dataset1	Dataset2	Dataset3	Dataset4	Dataset5	Dataset6
All identified irlncRNAs							
Count	7,038	1,173	1,173	1,173	1,173	1,173	1,173
Min	0.028 1	0.345 5	0.298 8	0.263 2	0.231 8	0.197 7	0.028 1
Max	0.558 7	0.558 7	0.345 4	0.298 8	0.263 1	0.231 8	0.197 7
Mean	**0.271 3**	**0.399 9**	**0.320 2**	**0.280 6**	**0.247 6**	**0.215 4**	**0.164 2**
In LncRNADisease2.0							
Count1	613	202	111	102	63	61	74
Count1/Count	0.087 1	0.172 2	0.094 6	0.087	0.053 7	0.052	0.063 1
Min1	0.108 7	0.346	0.299	0.263 3	0.232 3	0.197 8	0.108 7
Max1	0.558 7	0.558 7	0.345 3	0.298 6	0.262 8	0.231	0.197 6
Mean1	**0.308 2**	**0.415 5**	**0.322 6**	**0.279 7**	**0.248 3**	**0.213 9**	**0.161 6**
In Lnc2Cancer2.0							
Count2	666	181	115	114	93	77	86
Count2/Count	0.094 6	0.154 3	0.098	0.097 2	0.079 3	0.065 6	0.073 3
Min2	0.091 5	0.345 5	0.299	0.263 3	0.231 8	0.197 9	0.091 5
Max2	0.558 7	0.558 7	0.345 3	0.298 6	0.263 1	0.231 6	0.196 8
Mean2	**0.295 9**	**0.411 1**	**0.321 3**	**0.280 8**	**0.249**	**0.214 5**	**0.163 3**

**TABLE 4:** The pathogenicity distribution of immune-related lncRNA (irlncRNA). The data set is divided equally according to the pathogenicity level of irlncRNAs.

Pathogenicity level of IrlncRNA	Total	Dataset1	Dataset2	Dataset3	Dataset4	Dataset5
All identified irlncRNAs						
Count	7,038	156	1,000	3,212	2582	88
Min	0.028 1	0.452 6	0.346 5	0.240 3	0.134 3	0.028 1
Max	0.558 7	0.558 7	0.452 5	0.346 4	0.240 3	0.133 8
Mean	**0.271 3**	**0.483 8**	**0.387 8**	**0.287 8**	**0.198 2**	**0.114 3**
In LncRNADisease2.0						
Count1	613	47	153	263	143	7
Count1/Count	0.087 1	0.301 3	0.153	0.081 9	0.055 4	0.079 5
Min1	0.108 7	0.452 6	0.346 5	0.240 5	0.135 5	0.108 7
Max1	0.558 7	0.558 7	0.451 7	0.346 4	0.240 2	0.132 8
Mean1	**0.308 2**	**0.490 6**	**0.393 3**	**0.293 2**	**0.193 5**	**0.126 1**
In Lnc2Cancer2.0						
Count2	666	36	143	300	177	10
Count2/Count	0.094 6	0.230 8	0.143	0.093 4	0.068 6	0.113 6
Min2	0.091 5	0.452 6	0.346 5	0.240 9	0.141 2	0.091 5
Max2	0.558 7	0.558 7	0.45	0.346	0.240 2	0.133 7
Mean2	**0.295 9**	**0.488 4**	**0.392 6**	**0.290 5**	**0.197 8**	**0.121 7**

## 4 Discussion

Long non-coding RNA plays an essential role in cancer via gene expression and immune regulation (Hu et al., 2013; Chen et al., 2017), and the identification of irlncRNA is of great significance to the diagnosis and treatment of cancer. This study implemented an R program, ImReLnc, to identify irlncRNAs and analyze their pathogenicity levels across cancer types. Firstly, ImReLnc calculated heuristic correlation based on direct correlation and partial correlation to provided a ranking score for lncRNA-related mRNA class. Then, ImReLnc performed immune-related enrichment analysis based on the ranking score mentioned above to obtain irlncRNAs. Besides, ImReLnc conducted differential expression analysis of these irlncRNAs. Finally, the pathogenicity levels of the irlncRNAs were estimated by their immune and differential expression characteristics across cancer types.

We compared the expression levels of irlncRNAs in immune cells and non-immune cells, which showed that the expression of irlcRNAs in immune cells was higher than that in non-immune cells (Figure 7). Besides, we analyzed the correlation between lncRNA expression and the level of immune infiltration. We found that the IR was significantly lower than the IRINF, and the INFR was also significantly lower than the INFRI, which indicated that the immunomodulatory effect of the identified irlncRNAs was significant (See Supplementary Table S3, Data Sheet 1). Furthermore, for the irlncRNAs in each cancer identified by ImReLnc, we compared them with those identified by ImmLnc (See Supplementary Table S5, Data Sheet 1). We found a significant overlap between the irlncRNAs recognized by the two methods, which further demonstrated the reliability of ImReLnc in recognizing irlncRNAs. Finally, we explored the application of ImReLnc in evaluating the pathogenicity level of irlncRNAs. For the pathogenicity levels of irlncRNAs estimated by ImReLnc, we compared them with the drlncRNAs in the disease database (Table 3 and Table 3), and the results showed that drlncRNAs had a significantly higher proportion in the highly pathogenic irlncRNA interval. We also compared the relationship between drlncRNA (from the LncRNADisease and Lnc2Cancer disease databases) and irlncRNA (identified by ImmLnc and ImReLnc), and we found that the irlncRNA recognized by ImReLnc accounted for a higher proportion in the disease database (Table 2). This indicates that our calculation of the pathogenicity levels of irlnRNAs is credible.

LncRNA FEZF1-AS1 was found to have the highest pathogenic level in cancer and was related to the occurrence of GBM, LUAD, HNSC, LGG, LUSC, PRAD, SKCM, BLCA, LIHC, READ, TGCT, THYM, ACC, MESO, UCS, and CHOL. We infer that lncRNA FEZF1-AS1 plays an important regulatory role in these 16 cancers. For GBM, LUAD, PRAD, LIHC, READ, and UCS, studies have shown that lncRNA FEZF1-AS1 plays a crucial regulatory role in them (Luo et al., 2020; Liu et al., 2018; Zhu et al., 2019; Wang et al., 2018; Bian et al., 2018; Zhang and Li, 2018). As for the other ten types of cancer, their cancer tissues are similar to or adjacent to the cancer tissues of the above six types of cancer (See Supplementary Table S6, Data Sheet 1). Studies have shown that cancers with similar original tissues may share lncRNA regulatory mechanisms (Zhang et al., 2016; Li et al., 2020). The original tissue of LGG is similar to GBM, the original tissue of LUSC and MESO is similar to LUAD, and the original tissue of CHOL is similar to LIHC. Therefore, like in their similar tissues, lncRNA FEZF1-AS1 may also play an immunoregulatory role in LGG, LUSC, MESO, and CHOL. The original tissue of THYM and HNSC is adjacent to GBM, the original tissue of ACC is adjacent to GBM, the original tissue of BLCA and TGCT is adjacent to PRAD, and the original tissue of SKCM is adjacent to UCS. These cancers may be caused by the metastasis of their adjacent tissues. Thus they have a similar irlncRNA regulatory mechanism as their adjacent tissues. Among them, ACC and SKCM are relatively far from their neighboring tissues, which may be caused by long-distance metastasis of advanced cancer. Overall, lncRNA FEZF1-AS1 is highly pathogenic and plays a crucial immunomodulatory role in cancer, which can provide a necessary reference for cancer treatment.

In summary, ImReLnc accurately identified irlncRNAs in cancers based on heuristic correlation optimization. More importantly, ImReLnc effectively assessed the pathogenicity level of irlncRNAs based on their immune and differential expression characteristics. However, due to the limitation of data acquisition, ImReLnc can only identify irlncRNAs in 33 cancers. In addition, we identified genes related to lncRNA expression and further investigated the enriched immune pathways, but it is still challenging determine how specific lncRNA affects gene expression. In the future, we hope to perform irlncRNA recognition for other types of cancer and explore the targets of these irlncRNAs.

## Data Availability

Publicly available datasets were analyzed in this study. This data can be found here: (1) TCGA: https://portal.gdc.cancer.gov/, (2) GENCODE: https://www.gencodegenes.org/, (3) PanglaoDB: https://panglaodb.se/index.html, (4) TIMER: http://cistrome.dfci.harvard.edu/TIMER/, (5) LncRNADisease2.0: http://www.rnanut.net/lncrnadisease/, (6) Lnc2Cancer2.0: http://www.bio-bigdata.net/lnc2cancer2.0/index.html, (7) Immport: https://www.immport.org/home.
