# Supplementary material for: ImReLnc: Identifying Immune-Related LncRNA Characteristics in Human Cancers Based on Heuristic Correlation Optimization
Source: Front Genet. 2022 Jan 10;12:792541. doi: 10.3389/fgene.2021.792541 (PMC8784420; doi:10.3389/fgene.2021.792541)
Supplement: Supplementary file 3 [file DataSheet1.PDF]

Supplementary Material

1 SUPPLEMENTARY FIGURES AND TABLES

1.1 Figures

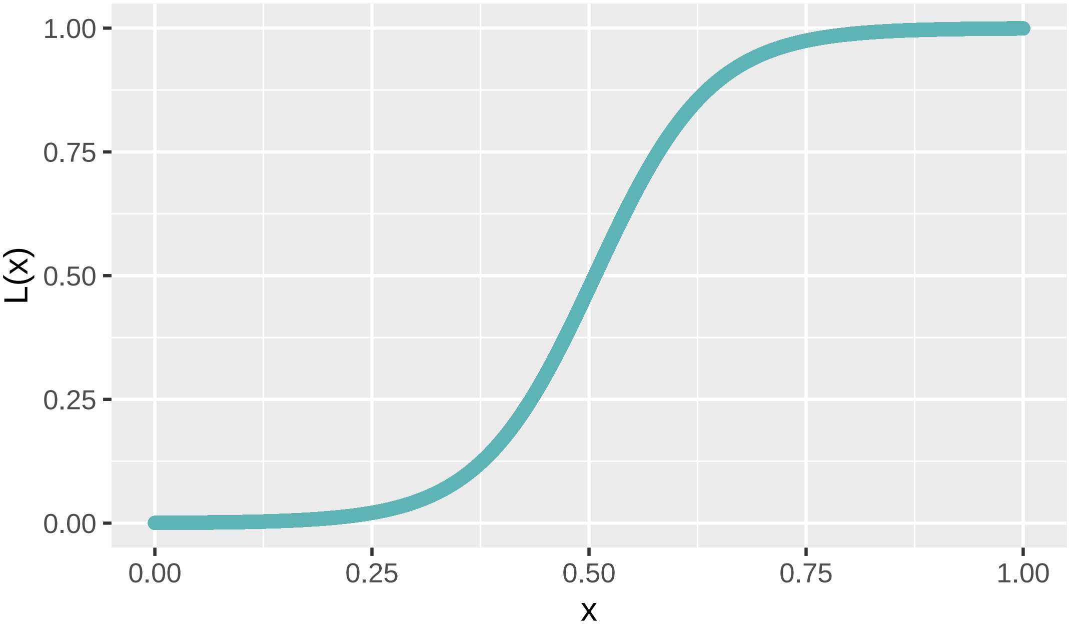

Figure S1. The distribution of logistic function.

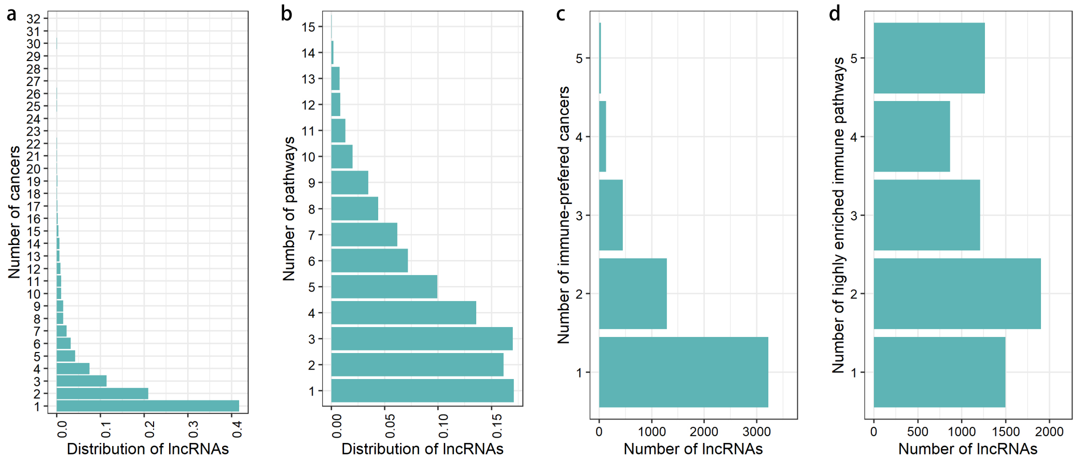

Figure S2. The number of immune-related lncRNAs (irlncRNAs) involved in different numbers of cancers and immune pathways **a** The number of irlncRNAs involved in different numbers of cancers. **b** The number of irlncRNAs involved in different numbers of immune pathways. **c** The number of irlncRNAs involved in different numbers of immune-prefered cancers. **d** The number of irlncRNAs involved in different numbers of highly enriched immune pathways.

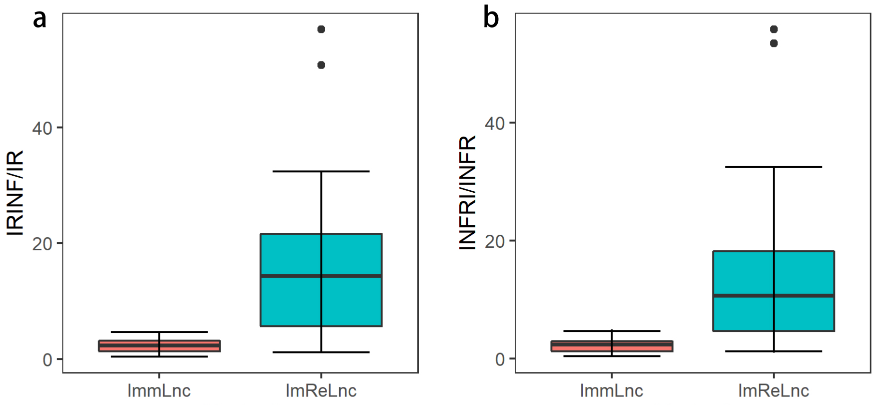

Figure S3. The comparison of the distribution of infiltration-related lncRNA (infrlncRNA) and immune-related lncRNA (irlncRNA) in ImReLnc and ImmLnc. IR refers to irlncRNA rate, IRINF refers to irlncRNA rate in infrlncRNA, INFR refers to infrlncRNA rate, and INFRI refers to infrlncRNA rate in IrlncRNA. **a** The distribution of IRINF/IR. **b** The distribution of INFRI/INFR.

1.2 Tables

Table S1. The distribution of cancer samples used in our analysis.

Cancer	Full name	Tumor	Normal	Total
ACC	Adrenocortical carcinoma	0	79	79
BLCA	Bladder Urothelial Carcinoma	19	414	433
BRCA	Breast invasive carcinoma	113	1109	1222
CESC	Cervical squamous cell carcinoma and endocervical adenocarcinoma	3	306	309
CHOL	Cholangiocarcinoma	9	36	45
COAD	Colon adenocarcinoma	41	480	521
DLBC	Lymphoid Neoplasm Diffuse Large B-cell Lymphoma	0	48	48
ESCA	Esophageal carcinoma	11	162	173
GBM	Glioblastoma multiforme	5	169	174
HNSC	Head and Neck squamous cell carcinoma	44	502	546
KICH	Kidney Chromophobe	24	65	89
KIRC	Kidney renal clear cell carcinoma	72	539	611
KIRP	Kidney renal papillary cell carcinoma	32	289	321
LAML	Acute Myeloid Leukemia	0	151	151
LGG	Brain Lower Grade Glioma	0	529	529
LIHC	Liver hepatocellular carcinoma	50	374	424
LUAD	Lung adenocarcinoma	59	535	594
LUSC	Lung squamous cell carcinoma	49	502	551
MESO	Mesothelioma	0	86	86
OV	Ovarian serous cystadenocarcinoma	0	379	379
PAAD	Pancreatic adenocarcinoma	4	178	182
PCPG	Pheochromocytomaand Paraganglioma	3	183	186
PRAD	Prostate adenocarcinoma	52	499	551
READ	Rectum adenocarcinoma	10	167	177
SARC	Sarcoma	2	263	265
SKCM	Skin Cutaneous Melanoma	1	471	472
STAD	Stomach adenocarcinoma	32	375	407
TGCT	Testicular Germ Cell Tumors	0	156	156
THCA	Thyroid carcinoma	58	510	568
THYM	Thymoma	2	119	121
UCEC	Uterine Corpus Endometrial Carcinoma	35	552	587
UCS	Uterine Carcinosarcoma	0	56	56
UVM	Uveal Melanoma	0	80	80

Table S2. The distribution of immune pathway-related gene lists used in our analysis.

Immune pathway	Number of enriched genes
Antigen_Processing_and_Presentation	146
Antimicrobials	505
BCRSignalingPathway	271
Chemokine_Receptors	52
Chemokines	102
Cytokine_Receptors	305
Cytokines	456
Interferons	3
Interferons_Receptor	17
Interleukins	47
Interleukins_Receptor	41
NaturalKiller_Cell_Cytotoxicity	134
TCRsignalingPathway	291
TGFb_Family_Member	33
TGFb_Family_Member_Receptor	12
TNF_Family_Members	12
TNF_Family_Members_Receptors respectively	19

Table S3. The distribution of infiltration-related lncRNA (infrlncRNA) and immune-related lncRNA (irlncRNA) in ImReLnc. IR refers to irlncRNA rate, IRINF refers to irlncRNA rate in infrlncRNA, INFR refers to infrlncRNA rate, and INFRI refers to infrlncRNA rate in IrlncRNA.

Cancer	Distribution of irlncRNA			Distribution of infrlncRNA		
	IR	IRINF	IRINF/IR	INFR	INFRI	INFRI/INFR
LIHC	0.01065695	0.60686869	56.945792	0.000499123	0.027848101	55.7940472
HNSC	0.01490625	0.75656566	50.754943	0.000520321	0.027795734	53.4203117
STAD	0.01510859	0.48987048	32.423302	0.001253429	0.020833333	16.6210762
BRCA	0.02509106	0.76974255	30.677965	0.00148388	0.045026882	30.3440249
OV	0.02138136	0.62405372	29.186815	0.000558864	0.018026138	32.2549766
LUAD	0.02522595	0.72804028	28.860762	0.001136999	0.030748663	27.0437098
PRAD	0.03365709	0.90934066	27.017805	0.00065522	0.017463498	26.652894
KIRC	0.03203831	0.733069	22.881013	0.001083153	0.019690402	18.1787854
UCEC	0.0072845	0.15731293	21.595569	0.001271897	0.011904762	9.3598485
COAD	0.00951032	0.2	21.029787	0.000512613	0.007092199	13.8353863
CESC	0.01976258	0.40682906	20.585828	0.000809389	0.017995656	22.2336333
SKCM	0.03068933	0.56917471	18.546339	0.002567416	0.049911379	19.4403129
ESCA	0.03675975	0.60674603	16.505719	0.000433601	0.006815203	15.7176758
LUSC	0.04067179	0.6691116	16.45149	0.002436102	0.026338894	10.8119014
LGG	0.03520842	0.55681818	15.814916	0.003065867	0.013409962	4.373954
KIRP	0.02907055	0.4173253	14.355603	0.001235544	0.01803417	14.5961323
SARC	0.03824363	0.54749051	14.315863	0.001146634	0.01972102	17.1990493
BLCA	0.02684473	0.3409934	12.702433	0.001150007	0.013693467	11.9072931
PAAD	0.03014974	0.34150373	11.326922	0.00262302	0.023365648	8.9079195
LAML	0.0762849	0.72683973	9.527963	0.001693721	0.017879949	10.5566067
READ	0.01288277	0.11241145	8.725718	0.000941221	0.008091385	8.5966881
DLBC	0.04890058	0.3791687	7.753869	0.001148101	0.006206897	5.4062307
PCPG	0.01639013	0.11624864	7.092602	0.000926301	0.009876543	10.6623517
THCA	0.05982733	0.42029851	7.025193	0.002668285	0.020563698	7.7067084
TGCT	0.14926481	0.8382642	5.615953	0.002522314	0.015100166	5.986631
MESO	0.03089168	0.16115095	5.216646	0.001424656	0.006496965	4.560374
GBM	0.03662485	0.17316017	4.727942	0.001780247	0.003739048	2.1002975
THYM	0.14703899	0.4468854	3.039231	0.00459553	0.021238532	4.6215628
UVM	0.07345204	0.22266598	3.031447	0.002842979	0.007591062	2.6701088
KICH	0.1131121	0.27947882	2.470813	0.003787879	0.010353987	2.7334526
UCS	0.02839606	0.06960059	2.451065	0.001682097	0.002520723	1.4985597
ACC	0.03197086	0.07298053	2.28272	0.002829724	0.002698459	0.9536122
CHOL	0.15924727	0.18609453	1.168589	0.008757442	0.013336081	1.5228283

Table S4. The distribution of infiltration-related lncRNA (infrlncRNA) and immune-related lncRNA (irlncRNA) in ImmLnc. IR refers to irlncRNA rate, IRINF refers to irlncRNA rate in infrlncRNA, INFR refers to infrlncRNA rate, and INFRI refers to infrlncRNA rate in IrlncRNA.

Cancer	Distribution of irlncRNA			Distribution of infrlncRNA		
	IR	IRINF	IRINF/IR	INFR	INFRI	INFRI/INFR
HNSC	0.16127074	0.75238095	4.6653283	0.00052032	0.00256916	4.9376365
SKCM	0.11587751	0.52766641	4.5536567	0.00256742	0.01237373	4.8195251
LUSC	0.17422096	0.72947054	4.1870423	0.00243610	0.00806176	3.3092874
KIRC	0.18764333	0.68751813	3.6639625	0.00108315	0.00298135	2.7524747
LIHC	0.18400108	0.66505051	3.6143837	0.00049912	0.00175953	3.5252437
SARC	0.17266963	0.60699685	3.5153653	0.00114663	0.00479403	4.1809617
STAD	0.20531499	0.71910541	3.5024497	0.00125343	0.00410644	3.2761648
PRAD	0.26352354	0.84492412	3.2062567	0.00065522	0.00201104	3.0692648
UCEC	0.12896263	0.41062425	3.1840560	0.00127190	0.00358637	2.8197033
LGG	0.15884257	0.47198369	2.9713929	0.00306587	0.00532716	1.7375694
LUAD	0.21185755	0.61242678	2.8907480	0.00113700	0.00341111	3.0000971
BRCA	0.25623904	0.72191007	2.8173305	0.00148388	0.00405809	2.7347807
KIRP	0.14427357	0.37810550	2.6207537	0.00123554	0.00276255	2.2358956
LAML	0.15904492	0.40952443	2.5748979	0.00169372	0.00475921	2.8099147
BLCA	0.16322676	0.41905071	2.5672917	0.00115001	0.00334711	2.9105111
OV	0.35289357	0.83775510	2.3739597	0.00055886	0.00136522	2.4428583
THCA	0.17847025	0.40963195	2.2952394	0.00266829	0.00678760	2.5438073
CESC	0.19674895	0.41267692	2.0974796	0.00080939	0.00190108	2.3487861
ESCA	0.22062593	0.45753968	2.0738255	0.00043360	0.00087348	2.0144706
TGCT	0.37940105	0.76217352	2.0088861	0.00252231	0.00514074	2.0381047
DLBC	0.16086605	0.26394554	1.6407785	0.00114810	0.00136724	1.1908721
THYM	0.27229192	0.44375702	1.6297106	0.00459553	0.01128726	2.4561386
COAD	0.13199784	0.21333333	1.6161880	0.00051261	0.00081758	1.5949224
PAAD	0.31195198	0.43035633	1.3795596	0.00262302	0.00335536	1.2791957
PCPG	0.12916498	0.17033411	1.3187329	0.00092630	0.00194952	2.1046313
MESO	0.16639687	0.21892002	1.3156499	0.00142466	0.00159174	1.1172821
ACC	0.10501821	0.10518340	1.0015730	0.00282972	0.00123971	0.4381037
UVM	0.05375691	0.04387535	0.8161805	0.00284298	0.00207026	0.7282023
KICH	0.12403885	0.09754943	0.7864426	0.00378788	0.00165604	0.4371941
READ	0.10973965	0.07583889	0.6910801	0.00094122	0.00067050	0.7123761
GBM	0.21010387	0.11651617	0.5545646	0.00178025	0.00053505	0.3005457
CHOL	0.23526238	0.11285812	0.4797117	0.00875744	0.00431208	0.4923905
UCS	0.22096317	0.08767415	0.3967817	0.00168210	0.00055443	0.3296089

Table S5. Comparative analysis of ImmLnc and ImRelnc. IS refers to the intersection of ImmLnc and ImReLnc.

Cancer	ImmLnc	ImReLnc	IS	ImmLnc/IS	ImReLnc/IS
TGCT	5625	2213	1521	0.27040000	0.6873023
LIHC	2728	158	100	0.03665689	0.6329114
PRAD	3907	499	307	0.07857691	0.6152305
BRCA	3799	372	226	0.05948934	0.6075269
OV	5232	317	190	0.03631498	0.5993691
LUAD	3141	374	222	0.07067813	0.5935829
PAAD	4625	447	265	0.05729730	0.5928412
HNSC	2391	221	122	0.05102468	0.5520362
STAD	3044	224	122	0.04007884	0.5446429
THYM	4037	2180	1141	0.28263562	0.5233945
UCEC	1912	108	54	0.02824268	0.5000000
CESC	2917	293	142	0.04868015	0.4846416
THCA	2646	887	426	0.16099773	0.4802706
LUSC	2583	603	288	0.11149826	0.4776119
KIRC	2782	475	216	0.07764198	0.4547368
BLCA	2420	398	179	0.07396694	0.4497487
COAD	1957	141	62	0.03168114	0.4397163
SKCM	1718	455	195	0.11350407	0.4285714
ESCA	3271	545	229	0.07000917	0.4201835
UCS	3276	421	168	0.05128205	0.3990499
CHOL	3488	2361	923	0.26462156	0.3909360
SARC	2560	567	219	0.08554687	0.3862434
READ	1627	191	71	0.04363860	0.3717277
PCPG	1915	243	90	0.04699739	0.3703704
GBM	3115	543	201	0.06452648	0.3701657
LAML	2358	1131	415	0.17599661	0.3669319
MESO	2467	458	163	0.06607215	0.3558952
DLBC	2385	725	256	0.10733753	0.3531034
LGG	2355	522	157	0.06666667	0.3007663
KIRP	2139	431	119	0.05563347	0.2761021
ACC	1557	474	125	0.08028259	0.2637131
KICH	1839	1677	309	0.16802610	0.1842576
UVM	797	1089	112	0.14052698	0.1028466

Table S6. Comparative analysis of the relationship between FEZF1-AS1 and cancer.

Cancer	PubMed	Similar tisues	Neighboring tisues
GBM	32210625	LGG	THYM,HNSC
LUAD	29510777	LUSC,MESO	**ACC**
PRAD	31298365	-	BLCA,TGCT
LIHC	29957463	CHOL	-
READ	29914894	-	-
UCS	29917186	-	**SKCM**
